# Synthesis, Bioactivity, Pharmacokinetic and Biomimetic Properties of Multi-Substituted Coumarin Derivatives

**DOI:** 10.3390/molecules26195999

**Published:** 2021-10-02

**Authors:** Annita Katopodi, Evangelia Tsotsou, Triantafylia Iliou, Georgia-Eirini Deligiannidou, Eleni Pontiki, Christos Kontogiorgis, Fotios Tsopelas, Anastasia Detsi

**Affiliations:** 1 Laboratory of Organic Chemistry, Department of Chemical Sciences, School of Chemical Engineering, National Technical University of Athens, Heroon Polytechniou 9, Zografou Campus, 15780 Athens, Greece; annitakatopodi@mail.ntua.gr (A.K.); evaggelia.tsotsou@yahoo.gr (E.T.); 2 Laboratory of Inorganic and Analytical Chemistry, Department of Chemical Sciences, School of Chemical Engineering, National Technical University of Athens, Heroon Polytechniou 9, Zografou Campus, 15780 Athens, Greece; ftsop@central.ntua.gr; 3 Laboratory of Hygiene and Environmental Protection, Department of Medicine, Democritus University of Thrace, 68100 Alexandroupolis, Greece; triailio@mbg.duth.gr (T.I.); edeligia@med.duth.gr (G.-E.D.); ckontogi@med.duth.gr (C.K.); 4 Laboratory of Pharmaceutical Chemistry, School of Pharmacy, Faculty of Health Sciences, Aristotle University of Thessaloniki, 54124 Thessaloniki, Greece; epontiki@pharm.auth.gr

**Keywords:** coumarins, antioxidant activity, lipoxygenase inhibition, cytotoxicity, molecular docking, biomimetic chromatography

## Abstract

A series of novel multi-substituted coumarin derivatives were synthesized, spectroscopically characterized, and evaluated for their antioxidant activity, soybean lipoxygenase (LOX) inhibitory ability, their influence on cell viability in immortalized human keratinocytes (HaCaT), and cytotoxicity in adenocarcinomic human alveolar basal epithelial cells (A549) and human melanoma (A375) cells, in vitro. Coumarin analogues **4a**–**4f**, bearing a hydroxyl group at position 5 of the coumarin scaffold and halogen substituents at the 3-phenyl ring, were the most promising ABTS^•+^ scavengers. 6,8-Dibromo-3-(4-hydroxyphenyl)-4-methyl-chromen-2-one (**4k**) and 6-bromo-3-(4,5-diacetyloxyphenyl)-4-methyl-chromen-2-one (**3m**) exhibited significant lipid peroxidation inhibitory activity (IC_50_ 36.9 and 37.1 μM). In the DCF-DA assay, the 4′-fluoro-substituted compound **3f** (100%), and the 6-bromo substituted compounds **3i** (80.9%) and **4i** (100%) presented the highest activity. The 3′-fluoro-substituted coumarins **3e** and **4e**, along with 3-(4-acetyloxyphenyl)-6,8-dibromo-4-methyl-chromen-2-one (**3k**), were the most potent lipoxygenase (LOX) inhibitors (IC_50_ 11.4, 4.1, and 8.7 μM, respectively) while displaying remarkable hydroxyl radical scavenging ability, 85.2%, 100%, and 92.9%, respectively. In silico docking studies of compounds **4e** and **3k**, revealed that they present allosteric interactions with the enzyme. The majority of the analogues (100 μΜ) did not affect the cell viability of HaCaT cells, though several compounds presented over 60% cytotoxicity in A549 or A375 cells. Finally, the human oral absorption (%HOA) and plasma protein binding (%PPB) properties of the synthesized coumarins were also estimated using biomimetic chromatography, and all compounds presented high %HOA (>99%) and %PPB (60–97%) values.

## 1. Introduction

Heterocyclic compounds are an invaluable source of diverse structures with chemical, biomedical, and industrial significance, finding a wide range of applications [1,2,3]. Coumarins constitute a large class of naturally or synthetically occurring heterocyclic compounds, belonging to the benzopyrone family [4] and exerting a wide range of biological properties including antioxidant [5], anti-inflammatory [6], neuroprotective [7], and anticancer [8]. Their fused heterocyclic ring system renders them a privileged scaffold in the medicinal chemistry field [9]. This characteristic, in combination with their significant pharmacological profile, constitute the structural modification of the coumarin core as a constantly developing research field.

Coumarins present significant antioxidant properties through several mechanisms such as by free radical scavenging [10], stimulation of the activity of antioxidant enzymes, such as superoxide dismutase and catalase [11], and modulation of transcription factors involved in oxidative stress [12]. It is also well known that the accumulation of free radicals is associated with the pathogenesis and progression of several diseases, including chronic inflammation and cardiovascular diseases, and the development of malignancy and metastasis among others [13].

Lipoxygenases (LOXs) are non-heme iron-containing enzymes that catalyze the peroxidation of polyunsaturated fatty acids, such as arachidonic acid and linoleic acid [14], producing metabolites that are implicated in many pathological conditions [15]. Therefore, inhibitors of LOX have attracted attention as potential agents for the treatment of various diseases, including asthma, psoriasis, inflammatory, and allergic diseases; and lately as anticancer agents [16,17].

In this context, coumarins have exhibited remarkable inhibitory activity against different types of LOX isoenzymes. For instance, in the work of Zerangnasrabad et al. [18], a series of *O*-prenylated-3-acetyl coumarin derivatives exhibited potent soybean LOX-15 inhibitory activity. In our previous work a series of 3-aryl-5-substituted-coumarin analogues presented significant soybean LOX inhibitory activity along with cytotoxicity against human neuroblastoma SK-N-SH and HeLa adenocarcinoma cell lines [16]. Moreover, several natural coumarins, including esculetin, scopoletin, and umbelliprenin have also presented combined LOX inhibitory activity [19,20] and anticancer activity [21]. However, it is noteworthy that several pharmaceutical agents have low effectiveness due to poor absorption and poor bioavailability problems [22], and they cause severe side effects. Therefore, estimation of the pharmacokinetic properties of novel drugs is essential.

Estimation of the ADME (absorption, distribution, metabolism, elimination) properties of a candidate drug is crucial. Their early estimation drastically increases a drug’s chances of being launched. Βiomimetic chromatography provides rapid and experimentally-based estimations of ADME properties in early stages. Two types of biomimetic stationary phases are mainly used in drug discovery: columns containing immobilized plasma proteins and immobilized artificial membrane (IAM) columns. Biomimetic properties are the retention outcomes and are useful for early ADME property estimation [23,24,25].

As a continuation of our previous studies towards the synthesis and bioactivity evaluation of coumarin analogues [10,16,26], we present herein the synthesis, structural characterization, and in vitro evaluation of biological and biomimetic properties of 25 multi-substituted coumarin analogues. The antioxidant activity of the novel derivatives was assessed using several different methods. Soybean LOX inhibitory activity, cell viability in human epidermal keratinocyte cells, (HaCaT), and cytotoxicity in adenocarcinomic human alveolar basal epithelial cells (A549) and human melanoma (A375) cell lines were also evaluated. Their human oral absorption (%HOA) and plasma protein binding (%PPB) properties were estimated using biomimetic chromatography. Finally, in silico docking studies of the most potent LOX inhibitors were performed in soybean LOX, providing a useful interpretation of the experimental results.

## 2. Results and Discussion

### 2.1. Chemistry

**Scheme 1:**
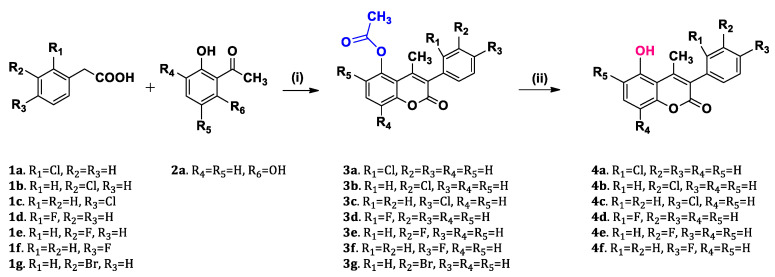
Synthetic route for 5-acetyloxy-coumarins (**3a**–**3g**) and 5-hydroxy coumarins (**4a**–**4f**) using: (i) acetic anhydride, Et_3_N, 120 °C, 3 h; and (ii) hydrazine monohydrate, methanol, 42 °C, 1–2 h.

**Scheme 2:**
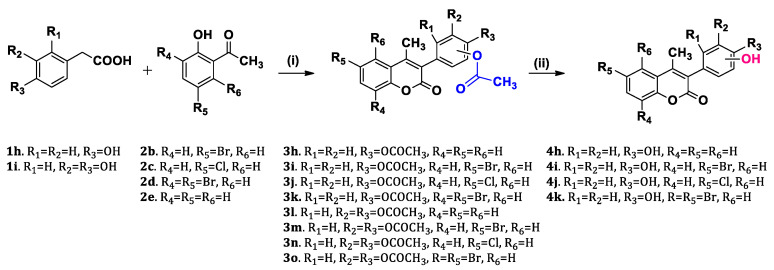
Synthetic route for 4′-acetyloxy or 3′,4′-diacetyloxy-coumarins (**3h**–**3o**) and 4′-hydroxy-coumarins (**4h**–**4k**) using: (i) acetic anhydride, Et_3_N, 120 °C, 3 h; and (ii) hydrazine monohydrate, methanol, 42 °C, 1–2 h.

As shown in Scheme 1 and Scheme 2, the acetyloxycoumarins (**3a**–**3o**) were synthesized first via a Perkin-Oglialoro condensation reaction between an appropriately substituted phenylacetic acid (**1a**–**1i**) and an appropriately substituted 2-hydroxyacetophenone (**2a**–**2e**) in acetic anhydride in the presence of a catalytic amount of triethylamine. As a result, seven 5-acetyloxycoumarins (**3a**–**3g**) and eight 4′-acetyloxy or 3′,4′-diacetyloxy-coumarins (**3h**–**3o**) were obtained. Subsequently, hydrazine monohydrate in methanol was used in order to remove the acetyl group and to produce the corresponding hydroxy-compounds, **4a**–**4f** and **4h**–**4k**.

### 2.2. Antioxidant Activity

Oxidative stress is a phenomenon caused by an imbalance between production and accumulation of reactive oxygen species (ROS) in cells and tissues, associated with several pathophysiological conditions, such as neurodegenerative diseases and cancer [27,28]. Antioxidants, which can be endogenous or exogenous, enzymatic or non-enzymatic, are essential defense mechanisms preventing the formation of free radicals, and neutralizing or repairing the damage caused by them [29].

It is well known that oxidative stress is a multifactorial phenomenon thus the antioxidant activity of a compound should be assessed via a variety of methods. In the present work all the coumarin analogues (Table 1) were evaluated for their antioxidant activity using four different in vitro assays.

Therefore, the antioxidant activity of the synthesized coumarins was evaluated using the following in vitro assays: (i) the 2,2-azino-bis(3-ethylbenzothiazoline-6-sulfonic acid (ABTS) radical cation (ABTS^•+^) reduction ability, (ii) the competition with DMSO for hydroxyl radical (HO^•^) scavenging ability, (iii) anti-lipid peroxidation ability, and (iv) the 2’,7’-dichlorofluorescein diacetate (DCFDA) assay, which was performed using the HaCaT cell line. The results are displayed in Table 2.

The ABTS radical cation scavenging assay is a decolorization assay frequently used as an antioxidant activity estimation protocol. Oxidation of ABTS salt with potassium persulfate generates the radical directly with no involvement of an intermediate radical, and the resulting solution has an intense blue color. Τhe presence of electron-donating antioxidants leads to the reduction of the radical and hence the discoloration of the solution, which is stoichiometric in relation to the amount of the active substance [30]. The absorbance is measured at 734 nm and the antioxidant capacity is compared to a standard antioxidant solution, such as ascorbic acid [31,32].

Regarding the ABTS radical scavenging assay, it is noted that none of the acetyloxy coumarin derivatives exhibited ABTS^•+^ inhibitory activity. However, the majority of the hydroxy coumarin analogues (**4a**–**4f**) presented significant ABTS^•+^ scavenging activity (>50%) at a concentration of 100 μΜ. The coumarin analogue **4d**, which has a fluoro substituent at position 2′ of the 3-phenyl ring and a hydroxyl group at position 5 of the coumarin scaffold, displayed the highest activity (73.3%). This could possibly be explained by the ability of phenolic compounds to release a proton from the OH group, which reacts with the ABTS^•+^ radical cation to form the colorless ABTS. However, it is noteworthy that substitution with a hydroxyl group at the 3-phenyl ring led to complete or significant loss of activity (**4h**–**4k**), indicating the importance of the presence of the hydroxyl group at position 5 of coumarin scaffold. Similar observations have been reported by the research group of Li et al. [33], who observed that hydroxyl substitution of the coumarin scaffold was important for increasing the antioxidant activity of the synthesized coumarin-tyrosol conjugates, and by Zhang et al. [34], who indicated that the presence of an OH moiety at the aromatic ring at position 4 of the coumarin core did not affect significantly the antioxidant activity of the 7,8-dihydroxy-4-phenyl-coumarin analogues.

The hydroxyl (HO^•^) free radical is considered as the most toxic and reactive radical among the ROS, leading to cellular or protein damage, membrane destruction, and even to cellular death when encountered in excess concentration [35]. In this context, the evaluation of the hydroxyl radical scavenging activity of the coumarin analogues was essential. Hydroxyl radicals are mainly produced by the Haber–Weiss reaction in the presence of iron or copper ions. In this protocol, iron-EDTA solution, which catalyzes the autooxidation of ascorbic acid, was used as the hydroxyl radical generation system. In general, the hydroxyl radicals formed oxidize the DMSO to form formaldehyde. The reaction involves the initial interaction of DMSO with the hydroxyl radicals to give methyl radicals, with the final product being formaldehyde, which is determined by Nash method. The intensity of the yellow solution formed was measured at 412 nm against a reagent blank [36,37].

All the coumarin derivatives were tested at a concentration of 100 μM, displaying significant scavenging activity against hydroxyl free radicals. Compounds **3d**, **3h**, **4b**, **4i**, **4j**, and **4k** exhibited 100% scavenging activity. It is interesting to note that although compound **3h**, bearing an acetyloxy substituent at position 4′ of the 3-aryl moiety, exhibited 100% HO^•^ scavenging activity, insertion of another acetyloxy moiety at the 3′ position of the phenyl ring (**3l**) led to complete loss of activity (10.7%), though its hydroxy analogue (**4h**) was also a potent scavenger (72.2%). Moreover, it is remarkable that compounds **4i**, **4j**, and **4k** possessing bromo or chloro substituents at the coumarin core along with a hydroxyl group at position 4′ of the 3-phenyl ring, exhibited 100% scavenging activity, indicating the importance of this substitution pattern for HO^•^ scavenging activity.

AAPH induced linoleic acid oxidation has been developed as a quick and reliable antioxidant activity assay. It is based on the inhibition of linoleic acid oxidation, initiated by the thermal free radical producer 2,2′-azobis(2-amidinopropane) dihydrochloride (AAPH), providing a measure of how efficiently antioxidants protect against lipid oxidation in vitro. Oxidation of exogenous linoleic acid by AAPH is followed by UV spectrophotometry at 234 nm in a highly diluted sample [38].

In the in vitro lipid peroxidation assay, it was observed that most of the acetyloxy coumarin derivatives presented satisfactory inhibitory activity. Compound **3m**, possessing a bromo substituent at position 6 of the coumarin scaffold along with two acetyloxy groups at positions 3′ and 4′ of the 3-aryl moiety, was the most potent inhibitor (IC_50_ 37.1 μM). It was also noted that the presence of a fluoro substituent on the 3-phenyl ring of the acetyloxy analogues led to inactive compounds (**3d**, **3e**, and **3f**). Regarding the hydroxy analogues, the most potent inhibitor was compound **4k**, bearing two bromo substituents on the coumarin scaffold (IC_50_ 36.9 μM), whereas it is noteworthy that its acetyloxy analogue (**3k**) was inactive. Coumarin derivative **3g**, bearing a bromo moiety at the 3′ position of the 3-phenyl group, is also a potent lipid peroxidation inhibitor with an IC_50_ of 50.7 μM, indicating that the presence of a bromo substituent enhances the anti-lipid peroxidation activity of the compounds, although its position on the coumarin scaffold plays an important role, as has also been reported in other studies [10,16].

In the setting of evaluating bioactive compounds in terms of antioxidant capacity in various cell lines, DCF-DA is the most widely used assay for the detection of intracellular H_2_O_2_ and oxidative stress. DCF-DA is cell-permeable and is hydrolyzed intracellularly to the DCFH carboxylate anion, which is retained in the cell. Similar results to the prior assays were observed: the 4′-fluoro-substituted compound **3f** and 6-bromo substituted compounds **3i** and **4i** were found to significantly eliminate the presence of ROS induced by the H_2_O_2_ treatment in HaCaT cells, after 24 h.

### 2.3. Lipoxygenase Inhibitory Activity

In humans, lipoxygenase plays a key role in the biosynthesis of leukotrienes, which are the proinflammatory mediators mainly released from myeloid cells. However, lipoxygenases (LOXs) and their metabolites, e.g., LTB4, are also associated with allergy, cell differentiation, and carcinogenesis. Thus, the development of novel inhibitors of lipoxygenases is important for the treatment of various diseases [17,38].

Soybean LOX is a plant enzyme which has been used widely as a prototype for studying the functional and structural properties of the homologous family of lipoxygenases [16], presenting satisfactory homology with the human 5-LOX [14]. Linoleic acid (LA) is a standard substrate for plant LOXs. In this protocol, all new molecules were tested as inhibitors of soybean LOX as an indication of their anti-inflammatory activity (Table 3).

In this series of compounds, the most potent lipoxygenase inhibitor was compound **4e** with IC_50_ 4.1 μM, bearing a fluoro substituent at position 3′ of the 3-phenyl ring, along with a hydroxyl moiety at position 5 of the coumarin scaffold. It is noteworthy that its acetyloxy analogue, **3e**, is also a potent LOX inhibitor with an IC_50_ value of 11.4 μM, indicating the importance of the presence of a fluoro substituent at position 3′ of the 3-aryl moiety. Substitution with a fluorine group at 2′ or 4′ positions of the 3-phenyl ring led to the inactive acetyloxy coumarins **3d** and **3f**. Among the hydroxy analogues **4d** and **4f**, only the 2′-fluoro substituted analogue (**4d**) presented significant LOX inhibitory activity (IC_50_ 31.6 μM).

Moreover, the acetyloxy coumarin **3k**, bearing two bromo substituents on the coumarin core, along with the hydroxy derivative **4a**, bearing a chloro group at position 2′ of the 3-phenyl ring also, displayed strong LOX inhibitory activities with IC_50_ values of 8.7 μM and 10 μM, respectively. On the contrary, their hydroxy and acetyloxy analogues, **4k** and **3a**, respectively, were found to be inactive.

### 2.4. Computational Studies—Docking Simulations with Soybean Lipoxygenase

#### Molecular Modeling of the Synthesized Derivatives in Soybean LOX

**Figure 1:**
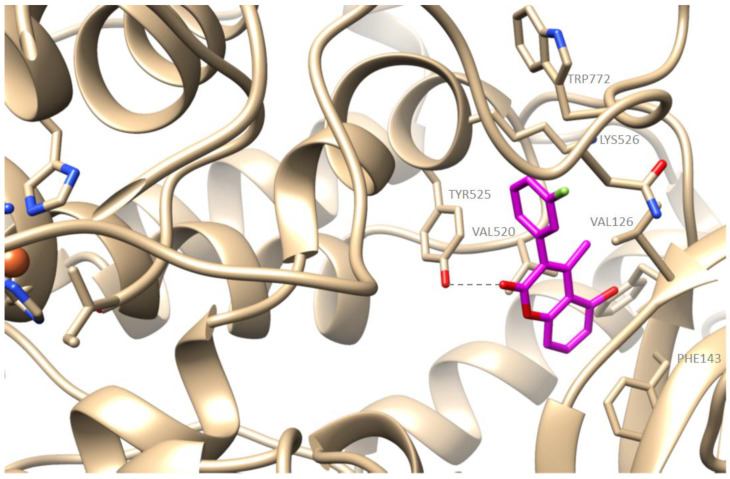
Preferred docking pose of **4e** (depicted in magenta) bound to soybean lipoxygenase (LOX-1).

**Figure 2:**
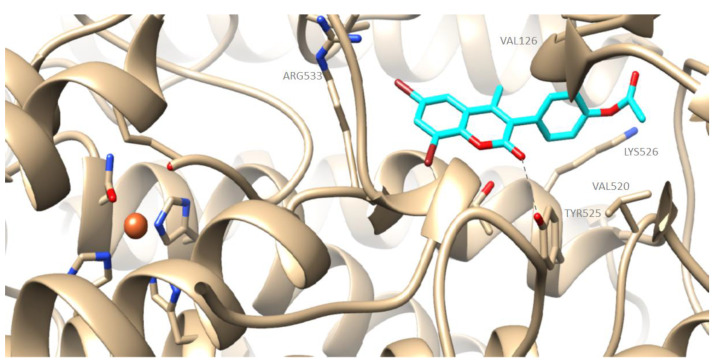
Preferred docking pose of **3k** (depicted in cyan) bound to soybean lipoxygenase (LOX-1).

All the synthesized derivatives have been studied in silico. The preferred docking poses of the most active derivatives, **4e** and **3k**, are shown in Figure 1 and Figure 2, respectively. Compound **4e** had an AutoDockVina score of −9.2 kcal/mol while **3k** –9.3 kcal/mol binding to soybean LOX (PDB code: 3PZW). Docking scores were obtained with algorithms and scoring function calculations. in vitro inhibition of soybean lipoxygenase was experimentally determined, so 100% correlation between the two evaluations could not be reached. Docking represents the preferred orientation of the ligand bound to the protein. It seems that the novel derivatives interact with the SLOX through allosteric interactions. Compound **4e** showed hydrophobic interactions with VAL126, PHE143, VAL520, TYR525, LYS526, and TRP772; a hydrogen bond with TYR525; and π-cation interactions with LYS526. Compound **3k** showed hydrophobic interactions with VAL126, VAL520, TYR525, LYS526, and ARG533; and a hydrogen bond with TYR525. It is well known that most LOX inhibitors act as antioxidants or scavenge free radicals, oxidizing the enzyme via a carbon-centered radical on a lipid chain [17]. It is possible that compounds **3k** and **4e** exert their activity by extending into the hydrophobic domain and blocking the substrates to the binding site, and thus preventing oxidation.

In the work of Lončarić et al. [39], it was also observed that substitution at position 6 of the coumarin scaffold with a bromine group led to potent soybean lipoxygenase inhibition. However, according to the molecular docking studies, the compounds of this study have interactions with the binding site of human lipoxygenase (5-LOX, in complex with arachidonic acid (PDB code: 3V99)) and soybean LOX (soybean LOX-3 in complex with (−)-epigallocatechin gallate ((−)-EGCG) (PDB code: 1JNQ)). Coumarin derivatives **4e** and **3k** seem to have allosteric interactions with soybean LOX (PDB code: 3PZW), as it has also been suggested in the literature [16].

### 2.5. Cell Viability in Human Epidermal Keratinocyte (HaCaT) Cell Line

The MTT assay (3- [4,5-dimethylthiazol-2-yl] -2,5 diphenyl tetrazole) is based on the conversion of MTT to formazan crystals by living cells, which determines mitochondrial activity. Since for most cell populations total mitochondrial activity is related to the number of viable cells, this assay is widely used to measure the in vitro cytotoxic effects of drugs on various cell lines or primary cells. The principle of the MTT assay is that for most viable cells the mitochondrial activity is stable, and therefore, an increase or decrease in the number of viable cells is linearly related to mitochondrial activity. The mitochondrial activity of the cells is reflected by the conversion of the MTT tetrazole salt to formazan crystals (bright purple), which can be solubilized (in this protocol with DMSO) for homogeneous measurement. Thus, any increase or decrease in the number of viable cells can be detected by measuring the formazan concentration reflected in the optical density (OD) measurement at 540 nm with a reference absorption of 720 nm.

All the compounds were tested for their cytotoxic activity against blank and control samples in the human epidermal keratinocyte line (HaCaT), at the concentration of 100 μΜ, and the results are presented in Table 4.

The synthesized coumarin derivatives showed low toxicity in the human epidermal keratinocyte line, though compounds **4c** and **4j** showed moderate cytotoxicity (63.7% and 73%, respectively) at the concentration of 100 μM. Compounds **4c** and **4j** both possess a chloro and a hydroxyl group in the coumarin framework, although at different positions; therefore, it is possible that this combination of substituents leads to increased cytotoxicity.

### 2.6. Evaluation of Coumarin Compounds’ Cytotoxicity against Cancer Cell Lines

Furthermore, the coumarin compounds synthesized in this study were also evaluated for their cytotoxicity against A549 (adenocarcinomic human alveolar basal epithelial cells) and A375 (human melanoma) cell lines. The results are presented in Table 5. It is worth noting that the evaluation of cell viability is a very delicate process which does not necessarily translate in all cell lines in the same way; hence, in this work we have evaluated three cell lines of interest in order to obtain a better view of the samples’ potential.

In the case of adenocarcinoma epithelial cells (A549), the hydroxy coumarins were found to present stronger cytotoxicity as opposed to their acetyloxy coumarin analogues. The compounds with a fluoro substituent (**4d** and **4f**) presented the highest toxicity rates (75.7% and 62.5%, respectively), followed by compounds with a bromo (**4k**, 66.5%) or chloro (**4a**, 64.7%) substituent.

On the contrary, almost all the coumarin compounds had moderate or low cytotoxicity against A375 melanoma cells. In this setting, the acetyloxy coumarin derivatives exhibited overall higher toxicity rates compared to the hydroxy coumarins. Compounds **3b** and **3e** presented the highest cytotoxicity, followed by **3i**, and **3j**, and **4b**. Similar results were obtained in our previous study [16], which indicated that the presence of an acetyloxy moiety on the coumarin scaffold significantly increases the cytotoxicity of the coumarin analogues against human neuroblastoma SK-N-SH and HeLa adenocarcinoma cell lines.

### 2.7. Physicochemical and Biomimetic Properties

The immobilized artificial membrane (IAM) chromatographic behavior of the 25 novel coumarin derivatives was studied. IAM stationary phases consisting of monolayers of phospholipids (e.g., phosphatidylcholine, PC) immobilized on a propylamine–silica support can simulate interactions of chemicals with cell membranes. It has been demonstrated that the IAM retention outcome is related to the permeability of chemicals, through membranes and retention factors can be used for the estimation with their intestinal absorption [25].

Using an IAM.PC.DD2 column (Regis Technologies) as the stationary phase with PBS as the mobile phase at pH = 7.40, and a flow rate of 3 mL/min in the presence of acetonitrile fractions (C%) of 10%, 15%, 20%, 25%, and 30%, a logk_C%_ value was obtained via the retention time for each concentration of CH_3_CN. Retention factors were then used to plot logk_C%_ as a function of acetonitrile fraction. The intercept of the linear regression corresponds to the logk_w_, corresponding to pure aqueous phase. IAM retention factors of the coumarin derivatives are presented in Table 5.

For the estimation of the percentages of the human oral absorption (%HOA) of the investigated compounds, the additional physicochemical properties required were calculated using the ADME Boxes version 3.0 software (PharmaAlgorithms). It can be observed that the compounds bearing a halogen substituent on the 3-phenyl ring and a hydroxyl group on the coumarin scaffold showed larger fractions of negative charge (F*-), and therefore, they were slightly acidic at pH values greater than 8.30 ± 0.80.

The %HOA values of the coumarin derivatives were obtained using Equation (1) [25] and are presented in Table 6.

(1) $$\%\;\mathrm{HOA}=\frac{100}{1+10^{-\left(2.17+0.88*\log k_{w}-0.006*\mathrm{MW}-0.83*A-0.53*F^{*+}+1.18*F^{*-}\right)}}$$

According to Bioclassification System (BCS) [40], a drug is considered highly permeable if the extent of absorption in humans is determined to be ≥ 90% of an administered dose. In this context, all the derivatives are likely to possess complete oral absorption, and they can therefore be considered suitable for use in pharmaceutical preparations intended for oral administration.

The results mentioned above are summed up in the following table.

The novel coumarin derivatives were also studied using the immobilized protein column HSA. An HSA chromatographic column provides estimates on the binding affinities of chemicals to human serum albumin, which is the most abundant plasma protein and is related to drug efficacy, drug—drug pharmacokinetic interactions, and drug safety [24]. HSA binding can be considered to represent almost the total plasma protein binding of a drug [25].

Retention factors on the HSA stationary phase were measured using as eluent PBS at pH = 7.00 and a flow rate of 1 mL/min in the presence of 10% acetonitrile. The obtained logk_10_ values are presented in Table 7.

The percentage of plasma protein binding (% PBB, Plasma Protein Binding) can be calculated using Equation (2) [24], and the results are displayed in Table 7.

(2) $$\%\;\mathrm{PPB}=101*\frac{10^{\log k_{10}}}{1+10^{\log k_{10}}}$$

As observed, the compounds exhibited high percentages of protein binding. High protein binding means that the drug is not flexible to diffuse and reach tissues, and it may undergo pharmacokinetic interactions with other drugs or endogenous substances (competition for the same protein binding sites). However, pharmacological action can increase in duration, while metabolism and renal excretion of the drug are slowed down, which may lead to a risk of drug accumulation and toxicity.

## 3. Materials and Methods

### 3.1. Chemicals and Instruments

The chemicals used for synthesis, analysis, and evaluation were purchased from Sigma-Aldrich and Fluka and used without further purification. NMR spectra were recorded on a Varian 300 and 600 MHz spectrometer and the HR-MS spectra on a UHPLC-MSn Orbitrap Velos-Thermo mass spectrometer at the National Hellenic Research Foundation. Melting points were obtained using a Gallenkamp MFB-595 melting point apparatus and are uncorrected.

### 3.2. Synthesis and General Procedures

#### 3.2.1. General Procedure for the Synthesis of Acetyloxy Coumarins (**3a**–**3o**):

A mixture of the appropriate phenylacetic acid (2.83 mmol) and the appropriate 2-hydroxyacetophenone (2.97 mmol) in acetic anhydride (3.1 mL) in the presence of triethylamine (8.77 mmol) was refluxed for 3 h. Water was then added, and the mixture was extracted with dichloromethane, dried over anhydrous Na_2_SO_4_, filtered, and concentrated in vacuo to afford the crude products, which on recrystallization from methanol and dichloromethane gave the purified products.

*5-acetyloxy-3-(2-chlorophenyl)-4-methyl-chromen-2-one* (**3a**), White solid, yield: 36%, m.p.: 136–137 °C, ^1^H NMR (600 MHz, CDCl_3_): δ (ppm) 7.54 (t, *J* = 8.4 Hz, HHHh1H, H-7), 7.52–7.50 (m, 1H, H-3′), 7.37–7.36 (m, 2H, H-4′, H-5′), 7.32 (dd, *J* = 8.4 Hz, *J* = 1.2 Hz, 1H, H-8), 7.24 (m, 1H, H-6′), 7.00 (dd, *J* = 8.4 Hz, *J* = 1.2 Hz, 1H, H-8), 2.35 (s, 3H, CH_3_-4), 2.29 (s, 3H, OCOCH_3_-5), ^13^C NMR (75 MHz, CDCl_3_): δ (ppm) 169.23, 159.17, 154.11, 148.14, 148.07, 134.25, 133.52, 131.51, 131.21, 130.06, 129.91, 127.25, 126.70, 119.98, 115.44, 114.17, 21.52, 19.89, HRMS calcd for C_18_H_12_O_4_Cl (M − H)^−^: *m*/*z*: 327.0502, found: 327.0422.

*5-acetyloxy-3-(3-chlorophenyl)-4-methyl-chromen-2-one* (**3b**), White solid, yield: 62%, m.p.: 150–153 °C, ^1^H NMR (600 MHz, CDCl_3_): δ (ppm) 7.53 (t, *J* = 8.4 Hz, 1H, H-7), 7.40–7.39 (m, 2H, H-5′, H-6′), 7.30 (dd, *J* = 8.4 Hz, *J* = 1.2 Hz, 1H, H-8), 7.27 (br, 1H, H-2′), 7.17–7.15 (m, 1H, H-4′), 6.99 (dd, *J* = 8.4 Hz, *J* = 1.2 Hz, 1H, H-6), 2.36 (s, 3H, CH_3_-4), 2.36 (s, 3H, OCOCH_3_-5), ^13^C NMR (75 MHz, CDCl_3_): δ (ppm) 169.24, 159.76, 153.80, 148.10, 147.12, 136.19, 134.53, 131.22, 130.15, 129.99, 128.67, 128.34, 127.74, 120.05, 115.35, 114.37, 21.50, 20.39, HRMS calcd for C_18_H_12_O_4_Cl (M − H)^−^: *m*/*z*: 327.0502, found: 327.0423.

*5-acetyloxy-3-(4-chlorophenyl)-4-methyl-chromen-2-one* (**3c**), White solid, yield: 34%, m.p.: 179–180 °C, ^1^H NMR (600 MHz, CDCl_3_): δ (ppm) 7.52 (t, *J* = 8.4 Hz, HHHh1H, H-7), 7.43 (d, *J* = 8.4 Hz, 2H, H-2′, H-6′), 7.30 (d, *J* = 8.4 Hz, 1H, H-8), 7.21 (d, *J* = 8.4 Hz, 2H, H-3′, H-5′), 6.99 (d, *J* = 8.4 Hz, 1H, H-6), 2.36 (s, 3H, CH_3_-4), 2.35 (s, 3H, OCOCH_3_-5), HRMS calcd for C_18_H_12_O_4_Cl (M − H)^−^: *m*/*z*: 327.0502, found: 327.0423.

*5-acetyloxy-3-(2-fluorophenyl)-4-methyl-chromen-2-one* (**3d**), White solid, yield: 24%, m.p.: 158–162 °C, ^1^H NMR (600 MHz, CDCl_3_): δ (ppm) 7.53 (t, *J* = 8.4 Hz, 1H, H-7), 7.43–7.40 (m, 1H, H-6′), 7.30 (dd, *J* = 8.4 Hz, *J* = 1.2 Hz, 1H, H-8), 7.28 (td, *J* = 7.2 Hz, *J* = 1.8 Hz, 1H, H-4′), 7.24 (t, *J* = 7.2 Hz, 1H, H-5′), 7.17 (t, *J* = 8.4 Hz, 1H, H-4′), 7.00 (dd, *J* = 8.4 Hz, *J* = 1.2 Hz, 1H, H-6), 2.36 (s, 3H, CH_3_-4), 2.36 (s, 3H, OCOCH_3_-5), HRMS calcd for C_18_H_12_O_4_F (M − H)^−^: *m*/*z*: 311.0798, found: 311.0718.

*5-acetyloxy-3-(3-fluorophenyl)-4-methyl-chromen-2-one* (**3e**), Off white solid, yield: 32%, m.p.: 150–152 °C, ^1^H NMR (600 MHz, CDCl_3_): δ (ppm) 7.53 (t, *J* = 7.8 Hz, 1H, H-7), 7.44–7.41 (m, 1H, H-5′), 7.30 (dd, *J* = 7.8 Hz, *J* = 1.2 Hz, 1H, H-8), 7.11 (td, *J* = 8.4 Hz, *J* = 2.4 Hz, 1H, H-4′), 7.05 (dt, *J* = 7.8 Hz, 1H, H-6′), 6.99 (dd, *J* = 7.8 Hz, *J* = 1.2 Hz, 2H, H-6, H-2′), 2.36 (s, 6H, OCOCH_3_-5, CH_3_-4), HRMS calcd for C_18_H_12_O_4_F (M − H)^−^: *m*/*z*: 311.0798, found: 311.0718.

*5-acetyloxy-3-(4-fluorophenyl)-4-methyl-chromen-2-one* (**3f**), White solid, yield: 30%, m.p.: 190–191 °C, ^1^H NMR (600 MHz, CDCl_3_): δ (ppm) 7.52 (t, *J* = 8.4 Hz, HHHh1H, H-7), 7.30 (d, *J* = 8.4 Hz, 1H, H-8), 7.26 (d, *J* = 6 Hz, 1H, H-6′), 7.24 (d, *J* = 5.4 Hz, 1H, H-2′), 7.15 (pseudotriplet, 2H, H-3′, H-5′), 6.99 (d, *J* = 7.8 Hz, 1H, H-6), 2.36 (s, 3H, CH_3_-4), 2.35 (s, 3H, OCOCH_3_-5), ^13^C NMR (75 MHz, CDCl_3_): δ (ppm) 169.38, 164.34, 161.05, 153.74, 147.99, 146.83, 132.01, 131.90, 131.09, 130.22, 128.06, 120.02, 115.94, 115.66, 115.39, 114.52, 21.56, 20.42, HRMS calcd for C_18_H_12_O_4_F (M − H)^−^: *m*/*z*: 311.0798, found: 311.0719.

*5-acetyloxy-3-(3-bromophenyl)-4-methyl-chromen-2-one* (**3g**), White solid, yield: 34%, m.p.: 133–135 °C, ^1^H NMR (600 MHz, CDCl_3_): δ (ppm) 7.54–7.51 (m, 2H, H-2′, H-7), 7.42 (s, 1H, H-6′), 7.33 (t, *J* = 7.8 Hz, 1H, H-3′), 7.29 (d, *J* = 8.4 Hz, 1H, H-8), 7.21 (d, *J* = 7.8 Hz, 1H, H-4′), 6.99 (d, *J* = 8.4 Hz, 1H, H-6), 2.35 (s, 6H, OCOCH_3_-5, CH_3_-4), ^13^C NMR (150 MHz, CDCl_3_): δ (ppm) 169.26, 159.78, 153.83, 148.11, 147.15, 136.46, 132.98, 131.60, 131.26, 130.26, 128.82, 127.69, 122.66, 120.07, 155.39, 114.37, 21.53, 20.44, HRMS calcd for C_18_H_12_O_4_Br (M − H)^−^: *m*/*z*: 370.9997, found: 370.9916.

*3-(4-acetyloxyphenyl)-4-methyl-chromen-2-one* (**3h**), Off white solid, yield: 27%, m.p.: 180–182 °C, ^1^H NMR (300MHz, CDCl_3_): δ (ppm) 7.69 (d, 1H, *J* = 7.8 Hz, H-5), 7.55 (t, 1H, *J* = 7.5 Hz, H-7), 7.39–7.32 (m, 4H, H-6, H-8, H-2′, H-6′), 7.19 (d, 2H, *J* = 7.8 Hz, H-3′, H-5′), 2.35 (s, 3H, CH_3_-4), 2.33 (s, 3H, OCOCH_3_-4′), HRMS calcd for C_18_H_13_O_4_ (M − H)^−^: *m*/*z*: 293.0892, found: 293.0814.

*3-(4-acetyloxyphenyl)-6-bromo-4-methyl-chromen-2-one* (**3i**), White solid, yield: 27%, m.p.: 185–186 °C, ^1^H NMR (600 MHz, CDCl_3_): δ (ppm) 7.79 (d, *J* = 2.4 Hz, 1H, H-5), 7.63 (dd, *J* = 9 Hz, *J* = 2.4 Hz, 1H, H-7), 7.31 (d, *J* = 8.4 Hz, 2H, H-2′, H-6′), 7.26 (d, *J* = 9 Hz,1H, H-8), 7.20 (d, *J* = 8.4 Hz, 2H, H-3′, H-5′), 2.32 (s, 3H, CH_3_-4), 2.32 (s, 3H, OCOCH_3_-4′), HRMS calcd for C_18_H_12_O_4_Br (M − H)^−^: *m*/*z*: 370.9997, found: 370.9917.

*3-(4-acetyloxyphenyl)-6-chloro-4-methyl-chromen-2-one* (**3j**), White solid, yield: 41%, m.p.: 194–195 °C, ^1^H NMR (600 MHz, CDCl_3_): δ (ppm)7.65 (d, *J* = 2.4 Hz,1H, H-5), 7.49 (dd, *J* = 9 Hz, *J* = 2.4 Hz, 1H, H-7), 7.32 (d, *J* = 8.4 Hz, 3H, H-8,H-2′, H-6′), 7.20 (d, *J* = 8.4 Hz, 2H, H-3′, H-5′), 2.33 (s, 3H, CH_3_-4), 2.32 (s, 3H, OCOCH_3_-4′), HRMS calcd for C_18_H_12_O_4_Cl (M − H)^−^: *m*/*z*: 327.0502, found: 327.0424.

*3-(4-acetyloxyphenyl)-6,8-dibromo-4-methyl-chromen-2-one* (**3k**), White solid, yield: 60%, m.p.: 240–241 °C, ^1^H NMR (600 MHz, CDCl_3_): δ (ppm)7.90 (d, *J* = 2.4 Hz,1H, H-7), 7.74 (d, *J* = 2.4 Hz, 1H, H-5), 7.31 (d, *J* = 8.4 Hz, 2H, H-2′, H-6′), 7.20 (d, *J* = 9 Hz, 2H, H- 3′, H-5′), 2.33 (s, 3H, CH_3_-4), 2.32 (s, 3H, OCOCH_3_-4′), ^13^C NMR (75 MHz, CDCl_3_): δ (ppm) 169.32, 159.21, 150.95, 148.73, 146.54, 137.02, 131.29, 131.10, 128.19, 127.25, 123.17, 232.85, 116.97, 111.58, 21.33, 17.05, HRMS calcd for C_18_H_11_O_4_Br_2_ (M − H)^−^: *m*/*z*: 448.9102, found: 448.9025.

*3-(4,5-diacetyloxyphenyl)-4-methyl-chromen-2-one* (**3l**), White solid, yield: 35%, m.p.: 182–184 °C, ^1^H NMR (600 MHz, CDCl_3_): δ (ppm) 7.69 (d, *J* = 7.8 Hz, HHHh1H, H-5), 7.55 (t, *J* = 7.2 Hz, 1H, H-7), 7.37 (d, *J* = 7.8 Hz, 1H, H-2′), 7.33 (t, *J* = 7.8 Hz, 1H, H-6), 7.29 (d, *J* = 8.4 Hz, 1H, H-3′), 7.23 (dd, *J* = 8.4 Hz, *J* = 1.8 Hz, 1H, H-8), 7.20 (d, *J* = 1.2 Hz, 1H, H-6′), 2.39 (s, 3H, CH_3_-4), 2.32 (s, 3H, OCOCH_3_-4′), 2.29 (s, 3H, CH_3_-5′), ^13^C NMR (75 MHz, CDCl_3_): δ (ppm) 168.13, 160.59, 152.76, 148.55, 142.02, 141.91, 132.74, 131.73, 128.54, 125.67, 125.35, 124.48, 123.39. 120.48, 116.97, 20.82, 20.79, 16.87, HRMS calcd for C_20_H_17_O_6_ (M + H)^+^: *m*/*z*: 353.0947, found: 353.1027.

*3-(4,5-diacetyloxyphenyl)-6-bromo-4-methyl-chromen-2-one* (**3m**), White solid, yield: 47%, m.p.: 190–191 °C, ^1^H NMR (600 MHz, CDCl_3_): δ (ppm) 7.80 (d, *J* = 1.8 Hz, 1H, H-5), 7.64 (dd, *J* = 8.4 Hz, *J* = 1.8 Hz, 1H, H-7), 7.29 (d, *J* = 8.4 Hz, 1H, H-3′), 7.25 (d, *J* = 7.8 Hz, 1H, H-8), 7.20 (dd, *J* = 7.8 Hz, *J* = 1.8 1H, H-2′), 7.18 (d, *J* = 7.8 Hz, 1H, H-6′), 2.36 (s, 3H, CH_3_-4), 2.32 (s, 3H, OCOCH_3_-5′), 2.30 (s, 3H, OCOCH_3_-4′), ^13^C NMR (75 MHz, CDCl_3_): δ (ppm) 168.10, 168.08, 159.93, 151.60, 147.33, 142.20, 141.95, 134.43, 132.20, 128.44, 127.99, 126.61, 125.59, 123.47, 122.17, 118.67, 117.23, 20.81, 20.78, 16.89, HRMS calcd for C_20_H_14_O_6_Br (M − H)^−^: *m*/*z*: 428.0052, found: 428.9970.

*3-(4,5-diacetyloxyphenyl)-6-chloro-4-methyl-chromen-2-one* (**3n**), White solid, yield: 40%, m.p.: 187–189 °C, ^1^H NMR (600 MHz, CDCl_3_): δ (ppm) 7.65 (s,1H, H-5), 7.50 (d, *J* = 9 Hz, 1H, H-7), 7.31 (d, *J* = 9.6 Hz, 1H, H-2′), 7.29 (d, *J* = 9 Hz, 1H, H-3′), 7.21 (d, 1H, H-8), 7.19 (s, 1H, H-6′), 2.36 (s, 3H, CH_3_-4), 2.32 (s, 3H, OCOCH_3_-5′), 2.30 (s, 3H, OCOCH_3_-4′), HRMS calcd for C_18_H_12_O_4_Cl (M − H)^−^: *m*/*z*: 385.0557, found: 385.0476.

*3-(4,5-diacetyloxyphenyl)-6,8-dibromo-4-methyl-chromen-2-one* (**3o**), White solid, yield: 65%, m.p.: 230–231 °C, ^1^H NMR (600 MHz, CDCl_3_): δ (ppm) 7.91 (d, *J* = 1.8 Hz, 1H, H-5), 7.74 (d, *J* = 1.2 Hz, 1H, H-7), 7.30 (d, *J* = 8.4 Hz, 1H, H-6′), 7.20 (dd, *J* = 8.4Hz, *J* = 1.8 Hz, 1H, H-2′), 7.17 (s, 1H, H-3′), 2.35 (s, 3H, CH_3_-4), 2.32 (s, 3H, OCOCH_3_-5′), 2.30 (s, 3H, OCOCH_3_-4′), ^13^C NMR (150 MHz, CDCl_3_): δ (ppm) 168.41, 159.21, 148.95, 147.31, 142.58, 142.22, 137.46, 132.06, 128.69, 127.55, 127.51, 125.84, 123.86, 123.26, 117.31, 111.86, 21.12, 21.11, 17.41, HRMS calcd for C_20_H_13_O_6_Br_2_ (M − H)^-^: *m*/*z*: 506.9157, found: 506.908.

#### 3.2.2. General Procedure for the Synthesis of Hydroxy Coumarins (**4a**–**4k**):

A mixture of the appropriate acetyloxy-coumarin (1.5 mmol) and hydrazine monohydrate (5 mmol per OH group) in methanol (24.3 mL) was stirred at 42 °C for 1–2 h. When TLC indicated the completion of the reaction, water was added and the mixture was extracted with ethyl acetate, dried over anhydrous Na_2_SO_4_, filtered, and evaporated. The crude products were triturated with methanol.

*3-(2-chlorophenyl)-5-hydroxy-4-methyl-chromen-2-one* (**4a**), White solid, yield: 91%, m.p.: >250 °C, ^1^H NMR (600 MHz, DMSO-*d*_6_): δ (ppm) 10.79 (br, 1H, OH-5), 7.59–7.58 (m, HHHh1H, H-2′), 7.45–7.43 (m, 3H, H-7, H-3′, H-5′), 7.37–7.36 (m, 1H, H-4′), 6.85 (d, *J* = 8.4Hz, 1H, H-8), 6.83 (d, *J* = 7.8 Hz, 1H, H-6), 2.33 (s, 3H, CH_3_-4), HRMS calcd for C_16_H_10_O_3_Cl (M − H)^−^: *m*/*z*: 285.0397, found: 285.0315.

*3-(3-chlorophenyl)-5-hydroxy-4-methyl-chromen-2-one* (**4b**), White solid, yield: 90%, m.p.: > 250 °C, ^1^H NMR (600 MHz, DMSO-*d*_6_): δ (ppm) 10.71 (br, 1H, OH), 7.48 (*J* = 8.4 Hz, 1H, H-7), 7.46–7.44 (m, 1H, H-2′), 7.40–7.38 (m, 2HHHh222H, H-5′, H-6′), 7.24 (d, *J* = 6.6 Hz, 1H, H-4′), 6.83 (d, *J* = 8.4 Hz, 1H, H-8), 6.82 (d, *J* = 9 Hz, 1H, H-6), 2.34 (s, 3H, CH_3_-4), ^13^C NMR (75 MHz, DMSO-*d*_6_): δ (ppm) 158.76, 157.20, 153.98, 151.04, 134.12, 133.40, 132.16, 132.09, 129.88, 129.26, 127.43, 122.49, 111.69, 108.69, 107.17, 20.77, HRMS calcd for C_16_H_10_O_3_Cl (M − H)^−^: *m*/*z*: 285.0397, found: 285.0314.

*3-(4-chlorophenyl)-5-hydroxy-4-methyl-chromen-2-one* (**4c**), White solid, yield: 70%, m.p.: >250 °C, ^1^H NMR (600 MHz, DMSO-*d*_6_): δ (ppm) 10.72 (br, 1H, OH-5), 7.50 (d, *J* = 7.8 Hz, 2HHHh22H, H-2′, H-6′), 7.39 (t, *J* = 8.4 Hz, 1H, H-7), 7.31 (d, *J* = 8.4 Hz, 2H, H-3′, H-5′), 6.82 (d, *J* = 7.8 Hz, 1H, H-8), 6.82 (d, *J* = 8.4 Hz, 1H, H-6), 2.40 (s, 3H, CH_3_-4), HRMS calcd for C_16_H_10_O_3_Cl (M − H)^−^: *m*/*z*: 285.0397, found: 285.0319.

*3-(2-fluorophenyl)-5-hydroxy-4-methyl-chromen-2-one* (**4d**), White solid, yield: 63%, m.p.: > 250 °C, ^1^H NMR (600 MHz, DMSO-*d*_6_): δ (ppm) 10.78 (br, 1H, OH), 7.49–7.46 (m, 1HHHh222H, H-6′), 7.42 (t, *J* = 7.8–8.4 Hz, 1H, H-7), 7.35 (t, *J* = 7.8 Hz, 1H, H-4′), 7.30–7.28 (m, 2H, H-3′, H-5′), 6.85 (d, *J* = 9.6 Hz, 1H, H-8), 6.83 (d, *J* = 9 Hz, 1H, H-6), 2.40 (s, 3H, CH_3_-4), HRMS calcd for C_16_H_10_O_3_F (M − H)^−^: *m*/*z*: 269.0692, found: 269.0612.

*3-(3-fluorophenyl)-5-hydroxy-4-methyl-chromen-2-one* (**4e**), Off white solid, yield: 61%, m.p.: > 250 °C, ^1^H NMR (600 MHz, DMSO-*d*_6_): δ (ppm) 10.71 (br, 1H, OH), 7.50–7.47 (m, 1HHHh222H, H-5′), 7.39 (t, *J* = 7.8–8.4 Hz, 1H, H-7), 7.22 (td, *J* = 9 Hz, *J* = 2.4 Hz, 1H, H-4′), 7.16 (d, *J* = 9 Hz, 1H, H-2′), 7.11 (d, *J* = 7.8 Hz, 1H, H-6′), 6.83 (d, *J* = 8.4 Hz, 1H, H-8), 6.81 (d, *J* = 9 Hz, 1H, H-8), 2.40 (s, 3H, CH_3_-4), HRMS calcd for C_16_H_10_O_3_F (M − H)^−^: *m/z*: 269.0692, found: 269.0610.

*3-(4-fluorophenyl)-5-hydroxy-4-methyl-chromen-2-one* (**4f**), Yellowish solid, yield: 82%, m.p.: >250 °C, ^1^H NMR (600 MHz, CDCl_3_): δ (ppm) 9.50 (br, 1H, OH-6), 7.16–7.12 (m, 3HHHh3333H, H-5, H-7, H-8), 7.00 (pseudotriplet, 2H, H-2′, H-6′),6.69 (d, *J* = 8.4 Hz, 1H, H-3′), 6.67 (dd, *J* = 8.4 Hz, *J* = 1.2 Hz, 1H, H-5′), 2.37 (s, 3H, CH_3_-4), ^13^C NMR (75 MHz, DMSO-*d*_6_): δ (ppm) 163.23, 159.99, 157.07, 153.77, 149.96, 132.53, 132.43, 131.81, 131.48, 123.85, 115.29, 115.01, 111.59, 109.15, 107.12, 21.38, HRMS calcd for C_16_H_10_O_3_F (M − H)^−^: *m*/*z*: 269.0692, found: 269.0610.

*3-(4-hydroxyphenyl)-4-methyl-chromen-2-one* (**4h**), White solid, yield: 72%, m.p.: > 250 °C, ^1^H NMR (600 MHz, DMSO-*d*_6_): δ (ppm) 9.61 (s, 1H, OH), 7.81 (d, *J* = 7.8 Hz, 1HHHh22H, H-5), 7.61 (t, *J* = 7.8 Hz, 1H, H-7), 7.40–7.37 (m, 2HHHh222H, H-6, H-8), 7.12 (d, *J* = 7.8 Hz, 2HHHh22H, H-2′, H-6′), 6.83 (d, *J* = 8.4 Hz, 2HHHh22H, H-3′, H-5′), 2.28 (s, 3H, CH_3_-4).

*6-bromo-3-(4-hydroxyphenyl)-4-methyl-chromen-2-one* (**4i**), White solid, yield: 42%, m.p.: >250 °C, ^1^H NMR (600 MHz, DMSO-*d*_6_): δ (ppm) 9.64 (br, 1H, OH-4′), 7.96 (s, HHHh1H, H-5), 7.77 (d, *J* = 9 Hz, 1H, H-7), 7.38 (d, *J* = 9 Hz, 1H, H-8), 7.12 (d, *J* = 8.4 Hz, 2H, H-2′, H-6′), 6.83 (d, *J* = 8.4 Hz, 2H, H-3′, H-5′), 2.27 (s, 3H, CH_3_-4).

*6-chloro-3-(4-hydroxyphenyl)-4-methyl-chromen-2-one* (**4j**), White solid, yield: 46%, m.p.: >250 °C, ^1^H NMR (600 MHz, DMSO-*d*_6_): δ (ppm) 9.64 (br, 1H, OH-4′), 7.85 (s, HHHh1H, H-5),7.65 (dd, *J* = 8.4 Hz, *J* = 2.4 Hz, 1H, H-7), 7.45 (d, *J* = 8.4 Hz, 1H, H-8), 7.12 (d, *J* = 8.4 Hz, 2H, H-2′, H-6′), 6.83 (d, *J* = 8.4 Hz, 2H, H-3′, H-5′), 2.28 (s, 3H, CH_3_-4).

*6,8-dibromo-3-(4-hydroxyphenyl)-4-methyl-chromen-2-one* (**4k**), Yellow solid, yield: 93%, m.p.: >250 °C, ^1^H NMR (600 MHz, DMSO-*d*_6_): δ (ppm) 9.69 (s, 1H, OH-4′), 8.15 (d, *J* = 1.8 Hz, 1H, H-7), 7.99 (d, *J* = 1.8 Hz, 1H, H-5), 7.13 (d, *J* = 9 Hz, 2H, H-2′, H-6′), 6.85 (d, *J* = 8.4 Hz, 2H, H-3′, H-5′), 2.28 (s, 3H, CH_3_-4), HRMS calcd for C_16_H_9_O_3_Br_2_ (M − H)^−^: *m/z*: 406.8997, found: 406.8920.

### 3.3. Evaluation of In Vitro Biological Activity

#### 3.3.1. ABTS Radical Scavenging Assay

For the evaluation of antioxidant activity, the scavenging capacities of all samples were measured via the ABTS assay, as previously described by Kontogiorgis et al. [41] with some modifications. Briefly, an ethanolic standard ABTS solution (7 mM) was prepared by the addition of ABTS salt and potassium persulfate. The solution was stored at 4–8 °C until it was bright blue and had an absorbance of 0.700±0.010 at 734 nm. Each sample was diluted in DMSO and tested in triplicate. The tested mixture contained 10–20 µL of sample and 990–980 µL of the ABTS solution, for a total volume of 1 ml. Blank samples contained equal amounts of DMSO instead of the coumarin analogues, and ascorbic acid was used as a reference sample for the final evaluation of the ABTS scavenging capacity. All mixtures were stored at room temperature to allow decolorization and measured after 6 min. The percentage reduction of the ABTS radical was evaluated according to the following Equation (3):

(3) $$\%\;\mathrm{Reduction}=\frac{\mathrm{Control}\;\mathrm{OD}\;\left(\mathrm{mean}\right)-\mathrm{Sample}\;\mathrm{OD}\;\left(\mathrm{mean}\right)}{\mathrm{Control}\;\mathrm{OD}\;\left(\mathrm{mean}\right)}\times 100$$

#### 3.3.2. Hydroxyl (HO^•^) Free Radical Scavenging Assay

For the evaluation of hydroxyl radical scavenging ability, the protocol described by Kontogiorgis et al. [37] was used. In short, each sample was tested in quadruplicate (A, B, C, D). For A and B, 10 μL of coumarin analogs diluted in DMSO (100 mM) were added; for C and D were the respective blanks. Standards (containing DMSO solution) and blanks were all prepared in a similar manner; to the latter an equal amount (10 µL) of solvent was added instead of the tested analogues. According to the protocol described by Klein et al., standard solutions of EDTA, FeCl_3_, ascorbic acid, and buffer solution (pH 7.4) were also added to each of the tested mixtures. All mixtures were incubated at 37 °C for 30 min, and then 1 mL of Nash solution and 250 mL of CCl_3_COOH solution were added. After a 10min incubation at 60 °C to allow the formation of the yellow mixture, the absorbance was measured at 734 nm.

The results were calculated as performance percentages in relation to the respective standards and blanks according to the following Equation (4):

(4) $$\%\;\mathrm{Scavenging}\;\mathrm{ability}=\frac{\;\mathrm{Mean}\;\mathrm{OD}\left(A+B\right)-\mathrm{Mean}\;\mathrm{OD}\left(C+D\right)}{\mathrm{Mean}\;\mathrm{OD}\left(A+B\right)}$$

#### 3.3.3. Inhibition of AAPH Induced Linoleic Acid Oxidation

For this experimental procedure, each coumarin analogue had a reference and a measurement sample. For the reference sample in a UV cuvette, 10 μL of the test sample (100 μM diluted in DMSO), 10 μL linoleic acid (LA) (16 mM), and buffer solution (pH 7.4) were added to a final volume of 1ml. For the measurement sample, in a UV cuvette, 10 μL of test sample, 50 μL AAPH (40 mM), 10 μL LA, and buffer solution were added to a final volume of 1 mL. Standard and blank samples were prepared in the same way using DMSO (solvent) instead of the tested coumarin analogues. The absorbance was measured after 2 min at 37 °C at 234 nm. Results were obtained via the following Equation (5):

(5) $$\%\mathrm{Inhibition}=\frac{\left(\mathrm{Standard}-\mathrm{Blank}\right)-\left(\mathrm{Reference}\;\mathrm{Sample}-\mathrm{Measurement}\;\mathrm{Sample}\right)}{\left(\mathrm{Standard}-\mathrm{Blank}\right)}\;\times \;100$$

#### 3.3.4. DCF-DA Assay Protocol

DCFDA is a fluorescent dye that measures the activity of hydroxyl radicals, peroxyl radicals, and other reactive oxygen species (ROS) within the cell. The DCFDA analysis protocol is based on the diffusion of DCFDA into the cell. It is then deacetylated from cellular esterases to a non-fluorescent compound, which is later oxidized by ROS to 2’,7’ -dichlorofluoroscein (DCF). The produced compound (DCF) is fluorescent and is detected by fluorescence spectroscopy at 485/535 nm (excitation/emission).

For the experimental procedure a standard solution of DCF-DA (20mM) in DMSO was prepared which was stored in the dark (−20 °C) until use. In a 96-well plate, cells were cultured to a density of 3 × 10^4^ cells/well.

The cells remained in the incubator (37 °C, 5% CO_2_) for 24 h, after which an appropriate amount of H_2_O_2_ (2 mM) was added and the dish was returned for incubation for the next 2 h. This was followed by the addition of the test substances (100μM) and incubation for 24 h. At the end of the treatment time, all wells were washed with PBS, and DCF-DA solution (working concentration: 10 μM) (in 200 μL of nutrient) was added and incubated for 40 min.

After incubation, the medium with the DCF-DA was removed and replaced with FBS-free DMEM. The measurement of the absorption was made immediately after that in a plate reader at 495/530 nm (excitation/emission).

All samples were tested in triplicates, against a control (without effects), blank (without cells), and positive control (standard 100 μM Ascorbic acid solution was used).

The percentage of antioxidant capacity (%) was calculated according to the following Equation (6) evaluating the presence of ROS [42]:

(6) $$\%\;AI=\frac{\left(\;\mathrm{OD}\;\mathrm{Sample}\;530\;\mathrm{nm}-\mathrm{Blank}\right)-(\mathrm{OD}\;\mathrm{Control}\;530\;\mathrm{nm}-\mathrm{Blank})}{\left(\mathrm{OD}\;\mathrm{Control}\;530\;\mathrm{nm}-\mathrm{Blank}\right)}\;\times \;100$$

#### 3.3.5. Inhibition of Soybean LOX

For this experimental procedure, a similar protocol to that of LA/AAPH was used in which each coumarin analogue had a reference and a measurement sample. For the reference sample in a UV cuvette 10 μL of the test sample (100 μM diluted in DMSO), 200 μL LOX solution (diluted in NaCl 0.9%), 100 μL LA (diluted in Tris buffer) and Tris buffer solution (pH 9) were added to a final volume of 1 mL. Respectively for the measurement sample, also in a UV cuvette 10 μL of test sample, 200 μL LOX solution, and Tris buffer solution (pH 9) were added to a final volume of 1mL. Standard and blank samples were prepared in the same way using DMSO (solvent) instead of the tested coumarin analogues. The absorbance was instantly measured at 234 nm and results were obtained via the following Equation (7):

(7) $$\%\mathrm{Inhibition}=\frac{\left(\mathrm{Standard}-\mathrm{Blank}\right)-\left(\mathrm{Reference}\;\mathrm{Sample}-\mathrm{Measurement}\;\mathrm{Sample}\right)}{\left(\mathrm{Standard}-\mathrm{Blank}\right)}\;\times \;100$$

#### 3.3.6. Cell Viability Assay

This protocol was used for the evaluation of cell viability and all the cell lines. It must be noted that this assay describes the count of cells that remain alive after the treatment. For the experimental procedure a standard solution of MTT 1mg/mL in PBS was prepared which after filtration (0.22 mm filter (Millipore, Carrigtwohill, Ireland)) was stored in the dark (4–8 °C) until use. In a 96-well plate, HaCaT, A549 and A375 cells were seeded in a number of 30 × 10^4^ cells/well for each evaluation series respectively. The cells were incubated (37 °C, 5% CO_2_) for 24 h, followed by the addition of the tested analogues (100 μM, diluted in DMEM) and another 24-h incubation. Next, appropriate amount of MTT was added in the tested wells and the plate returned to the incubator for the next 4 h for the formation of the formazan crystals. The formazan crystals were then redissolved by adding DMSO (100 μL) and stirring lightly for 40 min, after which the absorbance was measured in a plate reader at 540 nm with a reference absorbance of 720 nm.

All samples were tested in triplicate, against a control (only cells), blank (no cells), and positive control (100 μM standard silibinin solution was used as a “cell-killing” standard).

### 3.4. Computational Methods. Molecular Docking Studies on Soybean Lipoxygenase

For the docking studies, soybean lipoxygenase (PDB code: 3PZW) was used, and the visualization was accomplished through UCSF Chimera [43]. The protein was prepared: water molecules were removed, missing residues were added with Modeller [44], hydrogen atoms and AMBER99SB-ILDN charges were added, and the charge on iron was set to +2.0, with no restraint applied to the iron atom and the ligands. Open-Babel was used to generate and minimize ligand 3D coordinates using the MMFF94 force field [45]. Ligand topologies and parameters were generated by ACPYPE (Ante-ChamberPYthon Parser interfacE) [46] using Antechamber [47]. Energy minimizations were carried out using the AMBER99SB-ILDN force field [48] with GROMACS 4.6. Docking was performed with AutoDockVina (1.1.2) [49] applying a grid box of size 100 Å, 70 Å, 70 Å in X, Y, Z dimensions. The generation of docking input files and the analysis of the docking results was accomplished by UCSF-Chimera. Docking was carried out with an exhaustiveness value of 10 and a maximum output of 20 docking modes.

### 3.5. IAM and HSA Chromatographic Study

The physicochemical and biomimetic properties of the new coumarin analogues were studied using liquid chromatography (HPLC) in 220–230 nm Two types of chromatographic columns were used: (i) immobilized artificial membrane (IAM) (REGISIAM.PC.DD.2, Regis Technologies) column and (ii) human serum albumin (HSA) (CHIRAL PAK) column.

The training set consisted of 25 neutral and slightly acidic compounds. Their IAM retention factors were measured at pH 7.40 on a IAM.PC.DD.2 column. The mobile phase consisted of acetonitrile solvent (CH_3_CN) in various concentrations (10%, 15%, 20%, 25%, 30%) and a flow rate of 3 mL/min was selected. Their HSA retention factors were measured at pH 7.00 on HSA column. The mobile phase consisted of acetonitrile solvent (CH_3_CN) in 10% concentration and a flow rate of 1 mL/min was selected.

The different pH values (7.00 for HSA and 7.40 for IAM) were measured with an electronic pH meter MP125 and were achieved by preparing suitable buffers of phosphates and chlorides (0.12 g KH_2_PO_4_, 0.72 g Na_2_HPO_4_·2H_2_O, 4.00 g NaCl and 0.10 g KCl). Sodium citrate solution was used to determine the dead time, which was deducted from the results at the end of measurements. The elution time of each coumarin analogue was measured at least three times. Retention times were converted into logk values, which represent the logarithm of the retention indexes, using Equation (8).

(8) $$\log k=\log\left(\frac{t_{r}-t_{0}}{t_{0}}\right)$$

where *t_r_* is the retention time of the coumarin derivative and *t*_0_ is the dead time [50].

The predictions of the physicochemical properties of the new coumarin derivatives, including their theoretical lipophilicity values, logP, were calculated using the ADME Boxes version 3.0 software (PharmaAlgorithms).

## 4. Conclusions

In conclusion, twenty-five coumarin derivatives from which, to our knowledge, twenty-two are presented in the literature for the first time (except for compounds **4h**, **4i**, and **4j**), were synthesized, spectroscopically characterized, and evaluated for their biological and biomimetic properties. In particular, all the synthesized coumarins were evaluated for their antioxidant activity using different experimental methods, their soybean LOX inhibitory activity, their cell viability regarding HaCaT, and their cytotoxicity against A549 and A375 cell lines. The majority of the synthesized analogues were found to be non-toxic against the HaCaT cell line. On the contrary, the presence of toxicity after 24 h was observed in almost all the evaluated samples in A375 melanoma cells; the acetyloxy coumarin derivatives (**3b** and **3e** > 60%, **3i** and **3j** 50–60%) presented higher toxicity compared to the hydroxy coumarins. In the case of adenocarcinoma epithelial cells (A549), compounds **4d** and **4f** with fluoro substituents presented strong toxicity (75.7% and 62.5%, respectively), followed by bromo (**4k**, 66.5%) and chloro (**4a**, 64.7%) substituted compounds. Several compounds presented significant antioxidant activity, especially the hydroxy coumarin derivatives **4a**–**4f**, which exhibited promising ABTS^•+^ scavenging activity. Compounds **4k** and **3m** were the most potent lipid peroxidation inhibitors (IC_50_ 36.9 and 37.1 μM, respectively). In the DCF-DA assay, the 4′-fluoro-substituted compound **3f** (100%), and the 6-bromo substituted compounds **3i** (80.9%) and **4i** (100%), presented the highest activity. Moreover, coumarin analogues **4e** and **3k** were the most potent LOX inhibitors with IC_50_ 4.1 μM and 8.7 μM, respectively, and displayed significant hydroxyl radical scavenging ability, 100% and 92.9%. The molecular docking studies revealed that compounds **4e** and **3k** have allosteric interactions with the enzyme, and they are possibly exerting their activity by extending into the hydrophobic domain and blocking the substrates to the binding site, and thus preventing oxidation. It is noteworthy that compound **3e** also possesses combined LOX inhibition (IC_50_ 11.4 μM), HO^•^ inhibitory activity (85.2%), and cytotoxicity towards A375 melanoma cells (60.7%), indicating the importance of a 3′-fluoro substituent on the 3-phenyl ring of the coumarin scaffold. Finally, the chromatographic study of all the coumarins led to an estimation of complete (almost 100%) human oral absorption (%HOA), and to relatively high (60–97%) plasma protein binding (%PPB) results.

## Figures and Tables

**Table 1:** Substituents of the coumarin derivatives.

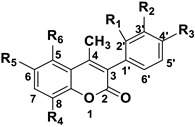						
Coumarin scaffold structure.						
Compound	R_1_	R_2_	R_3_	R_4_	R_5_	R_6_
**3a**	Cl	H	H	H	H	OCOCH_3_
**3b**	H	Cl	H	H	H	OCOCH_3_
**3c**	H	H	Cl	H	H	OCOCH_3_
**3d**	F	H	H	H	H	OCOCH_3_
**3e**	H	F	H	H	H	OCOCH_3_
**3f**	H	H	F	H	H	OCOCH_3_
**3g**	H	Br	H	H	H	OCOCH_3_
**3h**	H	H	OCOCH_3_	H	H	H
**3i**	H	H	OCOCH_3_	H	Br	H
**3j**	H	H	OCOCH_3_	H	Cl	H
**3k**	H	H	OCOCH_3_	Br	Br	H
**3l**	H	OCOCH_3_	OCOCH_3_	H	H	H
**3m**	H	OCOCH_3_	OCOCH_3_	H	Br	H
**3n**	H	OCOCH_3_	OCOCH_3_	H	Cl	H
**3o**	H	OCOCH_3_	OCOCH_3_	Br	Br	H
**4a**	Cl	H	H	H	H	OH
**4b**	H	Cl	H	H	H	OH
**4c**	H	H	Cl	H	H	OH
**4d**	F	H	H	H	H	OH
**4e**	H	F	H	H	H	OH
**4f**	H	H	F	H	H	OH
**4h**	H	H	OH	H	H	H
**4i**	H	H	OH	H	Br	H
**4j**	H	H	OH	H	Cl	H
**4k**	H	H	OH	Br	Br	H

**Table 2:** In vitro antioxidant evaluation of the synthesized compounds, through the (i) ABTS assay, (ii) the competition with DMSO for hydroxyl radical (HO^•^) scavenging ability, (iii) the inhibition of linoleic acid induced by AAPH assay *, and (iv) the DCFDA assay ^#,$^.

Compound	ABTS^•+^ % (100 μΜ)	HO^•^ % (100 μΜ)	AAPH IC_50_ (μΜ)/% (100 μΜ)	DCFDA Assay, % (100 μΜ)
**3a**	no	97.0	71.9	33.8
**3b**	1.9	96.4	91.5	56.9
**3c**	15.0	30.8	75.1	no
**3d**	no	100	42.6%	22.4
**3e**	2.0	85.2	no	no
**3f**	no	71.6	36.8%	100.0
**3g**	2.8	42.0	50.7	45.5
**3h**	no	100	70.7	no
**3i**	no	64.5	70.7	80.9
**3j**	no	26.6	no	9.0
**3k**	no	92.9	no	no
**3l**	2.8	10.7	85.5	no
**3m**	no	46.7	37.1	no
**3n**	no	65.7	84.3	no
**3o**	no	52.1	77.2	25.7
**4a**	56.5	37.3	no	0.0
**4b**	49.2	100	no	27.4
**4c**	65.2	48.5	86.6	no
**4d**	73.3	21.9	0.0%	no
**4e**	69.2	100	31.0%	no
**4f**	64.2	99.4	86.8	no
**4h**	8.6	72.2	no	no
**4i**	no	100	70.7	100
**4j**	no	100	36.1%	2.1
**4k**	27.8	100	36.9	no
Ascorbic acid	99.1	-	-	87.0
Trolox	92.0	85.0	92%	-

^*^ For the AAPH evaluation, results are presented either as IC_50_ values when available or as percentages (%) of inhibition at the compound concentration of 100μΜ. **^#^** The given results are from tests performed at least in triplicate, and each standard deviation is less than 10% of the mean. ^$^ No: no activity was determined in our experimental conditions.

**Table 3:** Soybean LOX in vitro inhibitory activity of the synthesized coumarin derivatives *^,#,$^.

Compound	IC_50_ (μM) / % (100 μΜ)
**3a**	17%
**3b**	31.6
**3c**	no
**3d**	11.6%
**3e**	11.4
**3f**	20.1%
**3g**	40.5
**3h**	33.8%
**3i**	46.5%
**3j**	49.5
**3k**	8.7
**3l**	31.4%
**3m**	48.0%
**3n**	56.5
**3o**	no
**4a**	10
**4b**	47.3%
**4c**	0.9%
**4d**	31.6
**4e**	4.1
**4f**	39.2%
**4h**	27.2%
**4i**	19.3%
**4j**	35.0%
**4k**	33.4%
NGDA	91%
Caffeic acid	600

* For the LOX evaluation, results are presented either as IC_50_ values when available or as percentages (%) of inhibition at the compound concentration of 100μΜ. ^#^ The given results are from tests performed at least in triplicate, and each standard deviation is less than 10% of the mean. ^$^ No: no activity was determined in our experimental conditions.

**Table 4:** Effects of the compounds on HaCaT cell line viability (%), at 100 μΜ.

Compound	Cell Viability % (100 μΜ)
**3a**	100
**3b**	100
**3c**	100
**3d**	100
**3e**	96.5
**3f**	100
**3g**	100
**3h**	96.2
**3i**	100
**3j**	90.5
**3k**	100
**3l**	100
**3m**	100
**3n**	100
**3o**	100
**4a**	100
**4b**	100
**4c**	63.7
**4d**	100
**4e**	92.4
**4f**	100
**4h**	100
**4i**	100
**4j**	73
**4k**	100
Silibinin	35.7

**Table 5:** Evaluation of coumarin compounds’ cytotoxicity against A549 and A375 cells, expressed as % of cell toxicity at 100 μΜ ^$^.

Compound	Cell Toxicity A549 % (100 μΜ)	Cell Toxicity A375 % (100 μΜ)
**3a**	no	38.7
**3b**	24.6	72.2
**3c**	no	24.8
**3d**	7.5	28.5
**3e**	8.9	60.7
**3f**	no	35.8
**3g**	no	7.02
**3h**	no	43.2
**3i**	no	51
**3j**	no	54.6
**3k**	no	28.8
**3l**	no	40.4
**3m**	no	33.3
**3n**	no	11.5
**3o**	no	45.6
**4a**	64.7	33.1
**4b**	no	52.8
**4c**	54.5	22.2
**4d**	75.7	11.5
**4e**	52.3	43.1
**4f**	62.5	45.4
**4h**	33.7	12.6
**4i**	21.7	6.48
**4j**	33.5	11.9
**4k**	66.5	no
Silibinin	78.3	84.4

^$^ No: no activity was determined in our experimental conditions.

**Table 6:** Physicochemical properties, chromatographic indexes, and estimated human oral absorption values (%HOA) for each compound using retention factors on the IAM.PC.DD.2 column and additional physicochemical properties (Equation (1) [25]).

			pH 7.40			
Compound	MW ^1^	logP ^2^	logk_w_	F*+ ^3^	F*− ^4^	%HOA
**3a**	328.7	4.42	3.58	0.000	0.000	100
**3b**	328.8	3.86	2.88	0.000	0.000	99.8
**3c**	328.7	4.42	3.05	0.000	0.000	99.9
**3d**	312.3	3.34	2.56	0.000	0.000	99.7
**3e**	312.3	3.34	2.47	0.000	0.000	99.7
**3f**	312.3	3.96	2.52	0.000	0.000	99.7
**3g**	373.2	4.08	4.77	0.000	0.000	100.0
**3h**	294.3	3.43	2.48	0.000	0.000	99.7
**3i**	373.2	4.59	3.28	0.000	0.000	99.8
**3j**	328.7	4.42	3.06	0.000	0.000	99.9
**3k**	452.1	5.36	3.91	0.000	0.000	99.9
**3l**	350.4	2.97	2.43	0.000	0.000	99.4
**3m**	431.2	4.20	3.21	0.000	0.000	99.6
**3n**	386.8	4.03	3.04	0.000	0.000	99.7
**3o**	510.1	4.96	3.79	0.000	0.000	99.6
**4a**	286.7	3.79	4.09	0.000	0.102	100
**4b**	286.7	3.79	3.73	0.000	0.102	99.9
**4c**	270.3	3.27	4.53	0.000	0.102	100
**4d**	270.3	3.27	4.69	0.000	0.102	100
**4e**	270.3	4.05	3.23	0.000	0.102	99.9
**4f**	252.3	3.07	2.62	0.000	0.007	99.7
**4h**	331.2	4.68	3.34	0.000	0.007	99.8
**4i**	286.7	4.51	3.23	0.000	0.007	99.9
**4j**	410.1	5.45	3.41	0.000	0.007	99.5

^1^ The exact molecular weight was calculated by Chem Draw Ultra 12.0 software. ^2^ Predicted logP values were calculated by ADME Boxes v. 3.0 software (Pharma Algorithms). ^3, 4^ Fractions of positively (F+) and negatively (F−) charged species were predicted by ADME Boxes version 3.0 software (Pharma Algoritmhs).

**Table 7:** HSA retention factors and estimated plasma protein binding (% PPB) of the investigated coumarin analogues.

	pH 7.00	
Compound	logk_10_	%PPB
**3a**	0.57	79.8
**3b**	0.80	87.2
**3c**	0.96	90.9
**3d**	0.73	85.1
**3e**	0.73	85.1
**3f**	0.57	79.4
**3g**	0.83	88.0
**3h**	0.74	85.5
**3i**	1.14	94.1
**3j**	0.97	91.2
**3k**	1.63	98.7
**3l**	0.16	59.9
**3m**	0.89	89.5
**3n**	0.74	85.5
**3o**	0.58	79.9
**4a**	0.79	87.0
**4b**	0.58	79.9
**4c**	0.72	84.8
**4d**	0.72	84.8
**4e**	1.34	96.6
**4f**	0.68	83.7
**4h**	1.38	97.0
**4i**	1.26	95.7
**4j**	0.59	80.4

## Data Availability

The data presented in this study are available on request from the corresponding author.

## Samples Availability

Samples of the compounds are available from the authors.
